# Teratological cases of the antennae in the family Aradidae (Hemiptera: Heteroptera)

**DOI:** 10.1038/s41598-020-57891-1

**Published:** 2020-01-23

**Authors:** Artur Taszakowski, Natalia Kaszyca-Taszakowska

**Affiliations:** University of Silesia in Katowice, Faculty of Natural Sciences, Institute of Biology, Biotechnology and Environmental Protection, Bankowa 9, 40-007 Katowice, Poland

**Keywords:** Developmental biology, Zoology

## Abstract

Teratological cases of the antennae in the family Aradidae (Hemiptera: Heteroptera) are widely described for the first time. Four hundred seventy-six specimens of flat bugs were studied, and antennal malformations were found in 14 of them (2.94%) (belonging to eight species and three subfamilies: Aradinae, Aneurinae and Mezirinae). All of the teratologies were observed using optical microscopy; moreover, in order to determine any compensatory regeneration, selected cases were also studied using a scanning electron microscope. In almost all of the specimens, the successful regeneration of the sensory organs to various degrees was observed. Additional results were the discovery of a previously unrecognized type of sensillum in flat bugs – a campaniform sensillum as well as significant differences in the distribution of the sensilla depending on the systematic affiliation.

## Introduction

Teratological cases of insects have long been of interest to entomologists. The first information about the abnormal structure of the Heteroptera antennae was given by Heineken in 1829^1^. He reported the existence of three-segmented antennae, which were shorter and thicker than those with a normal structure in the genus *Reduvius* (Reduviidae). Next, Burmeister provided information on the frequent collection of specimens of *Raglius alboacuminatus* (Rhyparochromidae) that had only three (but much larger) segments on one of the antennae^2^. The second half of the 19th century brought further data on the antennal teratology in various groups of the infraorder Pentatomomorpha: Alydidae, Blissidae, Coreidae, Cydnidae, Lygaeidae, Pentatomidae, Rhyparochromidae^3^, Cymidae^4^, Cimicomorpha – Miridae^5^ and in the Leptopodomorpha within the family Aepophilidae^6^. In the 20th century, cases of teratology were also documented in families of Pentatomomorpha such as Acanthosomatidae^7^, Aradidae and Scutelleridae^8^, Berytidae, Geocoridae and Rhopalidae^9^, Stenocephalidae^10^, Pyrrhocoridae^11^, Malcidae^12^, Heterogastridae^13^, Oxycarenidae^14^, Plataspidae, Thyreocoridae and Largidae^15^. In Gerromorpha, oligomery was observed in Gerridae and Vellidae^16^. Moreover, in Cimicomorpha, the regeneration of antennae was experimentally evidenced in Cimicidae. In specimens that had been collected in nature, anomalies were observed in the antennae in Anthocoridae^8^ and Tingidae, which were described in detail in an extensive paper by Štusák and Stehlík^15^. Recently, there have been many new reports on teratology cases in South American Heteroptera (e.g.^17,18^) including reports of the first malformation in the family Idiostolidae^19^. In addition to Heteroptera, the antennal malformations in other Hemiptera have only been well studied in the dwarfish males of the aphid genus *Stomaphis* Walker, 1870^20^.

Antennal anomalies can originate in the nymphal instars through injuries to the antennae and their subsequent regeneration during the next instars^15,21–23^. Antennal anomalies in Pentatomomorpha have been observed much more frequently than in other infraorders and they have also been found in a number of families that belong to this group^15,24,25^. Among Pentatomomorpha, oligomery (symphysomery), which depends on a reduction of the number of segments that results from an injury^14,15^, is the anomaly that has most often been reported. Regeneration is influenced by many factors, such as: the degree and type of injury, the developmental stage of nymph, the time before moulting, the condition of the specimens as well as the environmental factors^15^. The regeneration of antennae after they have been shortened was examined experimentally (e.g.^21–23,26,27^). The research on *Oncopeltus fasciatus* (Lygaeidae) showed, that after amputation of two or three antennal segments, the final number of segments was one less than in normally developed antenna. However, the remaining segments in that case grew abnormally larger and with bristle patterns characteristic of the last two antennal segments, suggesting compensation of the lost segments by excessive growth^23,26,27^. Such phenomenon was called “compensatory hyper-regeneration” by Wolsky^23^.

The functional interpretation of the regenerated antennal segments and their sensilla is very poorly understood – in periods of the greatest interest in teratological cases in Heteroptera, no satisfactorily technique was advanced. Despite the developments in imaging using scanning electron microscopy, not much research has been conducted using this technique. The only work using SEM concerned the compensatory regeneration of antennae after the removal of the distal segment in *Riptortus clavatus* (Alydidae)^27^.

The family Aradidae (flat bugs) includes eight subfamilies, about 200 genera and at least 2,000 currently known species, which range from 2.2 to 20 mm in size. Most Aradidae are flattened dorsoventrally and live on or under the bark of decaying trees and twigs or in debris on the floor of moist forests; they are always associated with fungal mycelia. Several species feed on the sap of dying or living trees^28^.

The Aradidae antennae are robust, sometimes very short, never very long, and are formed of four segments. The first segment is thick, the second and third are generally cylindrical or moniliform and the last one is more or less fusiform^29^. The antennal sensory organs of Aradidae are very poorly researched. Sinitsina and Chaika described the antennal sensilla of *Aradus corticalis annulicornis*. Six types of sensilla were identified: styloconic (S) that cover the entire surface of the antennae except for the small cone on the last segment where the other types of sensilla are located: sensilla chaetica (Ch1), trichoid (T1, T3) and basiconic (B1 + B2)^30^.

As was already mentioned, the first information on the abnormal antennal structure of Aradidae was presented by Štěpánek^8^. The fourth segment of the left antenna of *Aradus truncatus* Fieber, 1860, is forked in half of its length. In addition, reports on teratology in flat bugs can also be found in taxonomical papers. The holotype of *Mezira paraensis* Kormilev et Heiss, 1979, has an oligomeric right antenna^31^. Similarly, the left antenna of the holotype of *Sandakaptera hauseri* Vásárhelyi, 1988, is teratological and is three-segmented^32^.

Due to the small amount of information on antennal malformations in the family Aradidae, it might seem that this is a very rare phenomenon. However, even because holotypes with such teratologies are known, the hypothesis that this phenomenon is much more common in the flat bugs was proposed. Therefore, during inventory work on Aradidae in the collection of the Upper Silesian Museum in Bytom (USMB), special attention to was paid to the antennal malformation of these insects. All of these cases are presented in this work. Moreover, the almost complete lack of data prompted us to research the functions of the regenerated antennal segments and, in particular, the sensilla.

## Methods

### Materials examined

The study is based on dry material from the collection of the USMB (476 specimens of Aradidae). Fourteen teratological specimens of eight species belonging to three subfamilies of flat bugs were examined: *Aradus betulae* (Linnaeus, 1758), *Aradus betulinus* Fallén, 1807, *Aradus conspicuus* Herrich-Schäffer, 1835, *Aradus corticalis* (Linnaeus, 1758), *Aradus depressus* (Fabricius, 1794), *Aradus erosus* Fallén, 1807 (Aradinae); *Aneurus laevis* (Fabricius, 1775) (Aneurinae); *Brachyrhynchus membranaceus* (Fabricius, 1798) (Mezirinae).

### Light microscopy

In order to prepare high-quality photos that would enable advanced processing (obtaining a uniform background, easy mounting of parts of the images), the specimens were glued on to transparent entomological glue boards and then cleaned with a delicate brush. The color images of the antennae were captured using the following equipment: Leica M205C (stereomicroscope), Leica LED5000 HDI (high diffuse dome illumination), Leica DFC495 (camera), Leica application suite 4.9.0 (software), Image Composite Editor (panoramic image stitcher) and Adobe Photoshop CS6 graphic editor. In order to compare the construction of teratological and normal antennae, they have been presented in a linear form (created using the graphic editor).

### Scanning electron microscopy

The material was dissected (only antennae or whole heads) and cleaned in detergent using an ultrasonic cleaner after which a procedure following the method described by Kanturski *et al*. was applied: dehydration with the serial baths of 80%, 90% and 96% ethanol for 20 min each and two baths of 99.8% ethanol for 30 min each^33,34^. The antennae were glued with carbon adhesive discs on to the aluminium pin stubs, coated with a film of gold and photographed using a Phenom XL scanning electron microscope. The photographs were prepared using the Image Composite Editor (panoramic image stitcher) and the graphic editor Adobe Photoshop CS6.

### Terminology for the sensilla

The terminology and classifications of the sensilla follow Sinitsina and Chaika^30^ and Ahmad *et al*.^35^.

## Results

**Figure 1:**
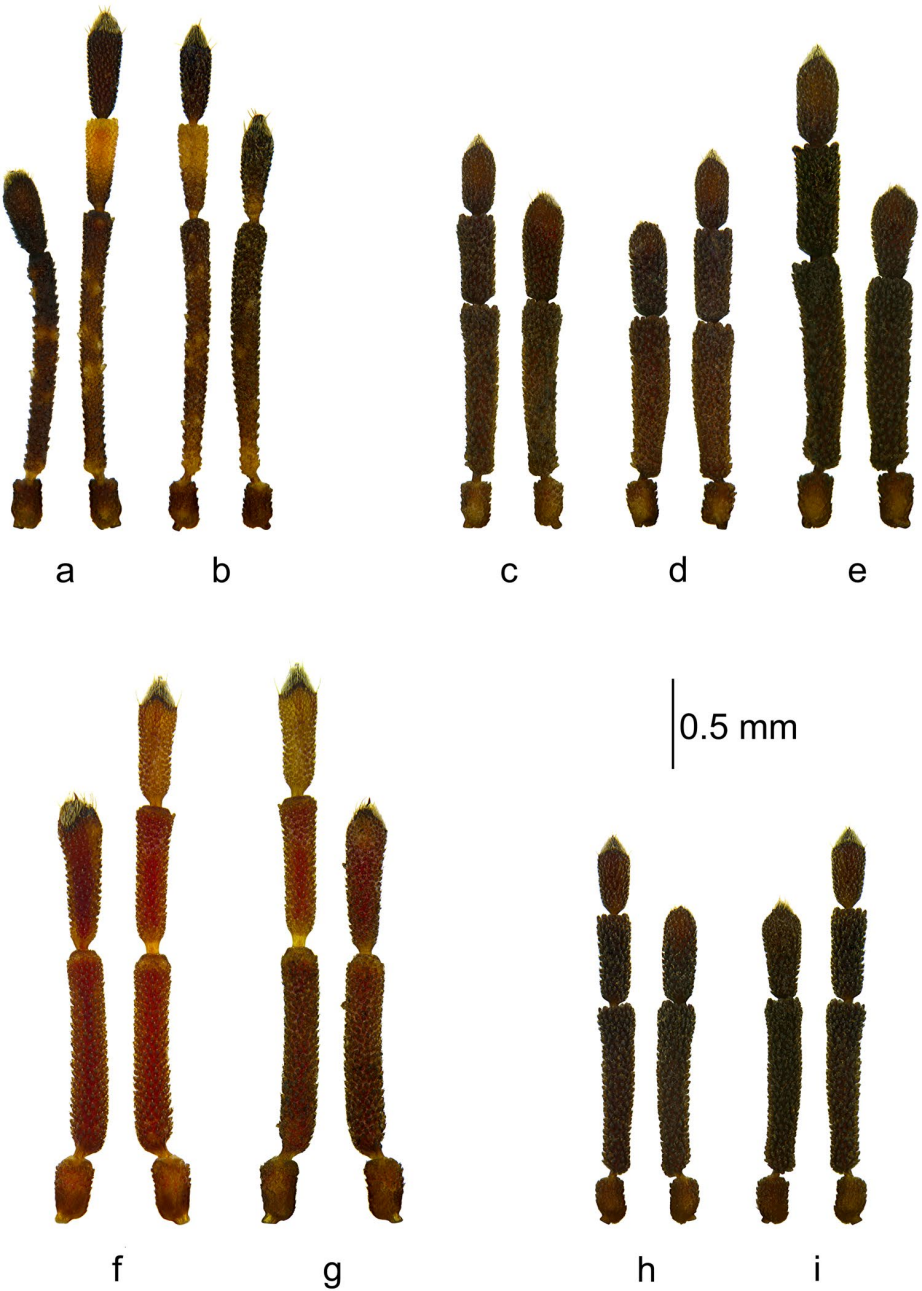
Pairs of antennae of the studied specimens: (**a**,**b**) *Aradus betulae*; (**c**–**e**) *Aradus betulinus*; (**f**,**g**) *Aradus conspicuous*; (**h**,**i**) *Aradus corticalis*.

**Figure 2:**
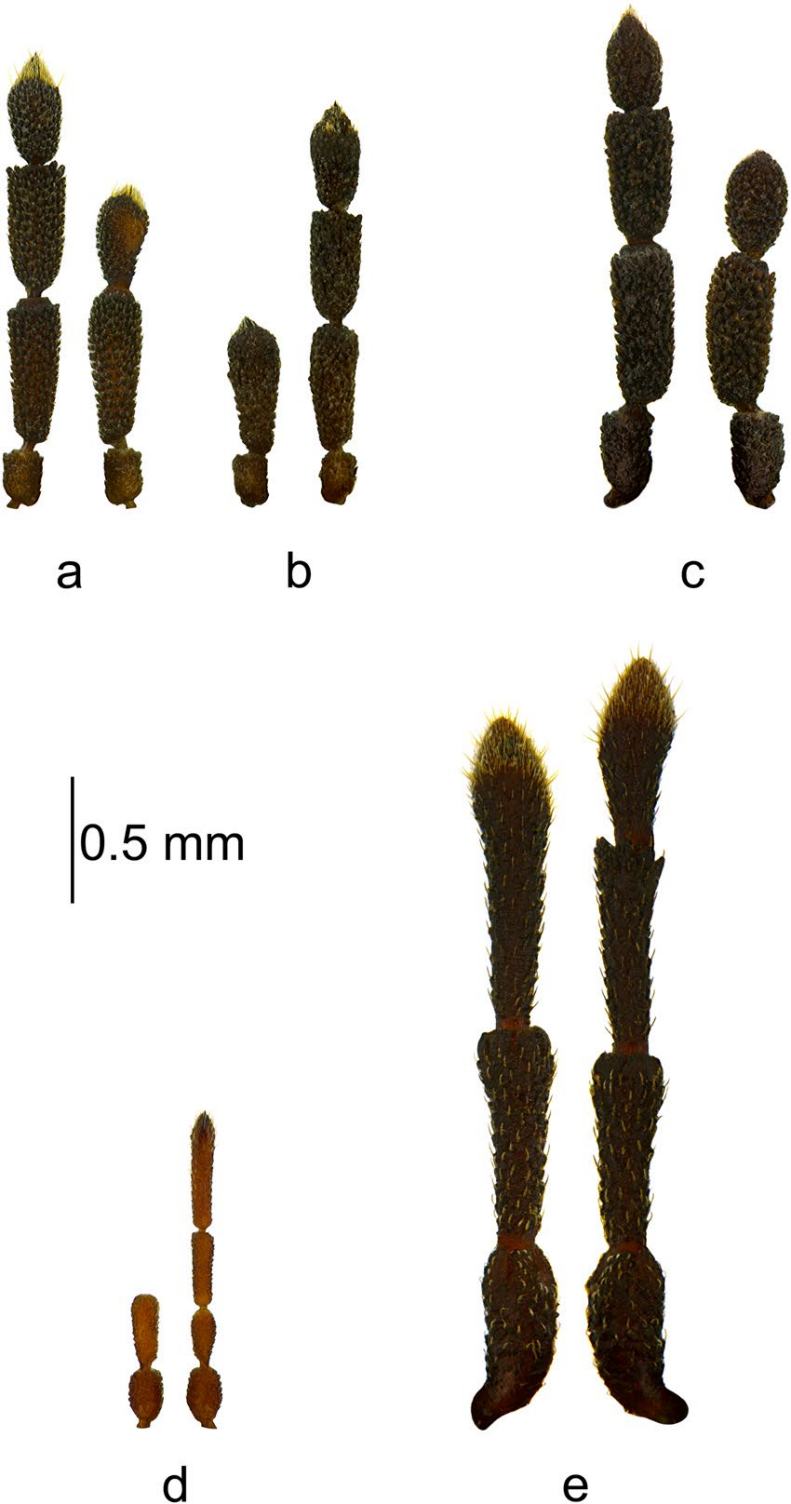
Pairs of antennae of the studied specimens: (**a**,**b**) *Aradus depressus*; (**c)** *Aradus erosus*; (**d**) *Aneurus laevis*; (**e**) *Brachyrhynchus membranaceus*.

**Figure 3:**
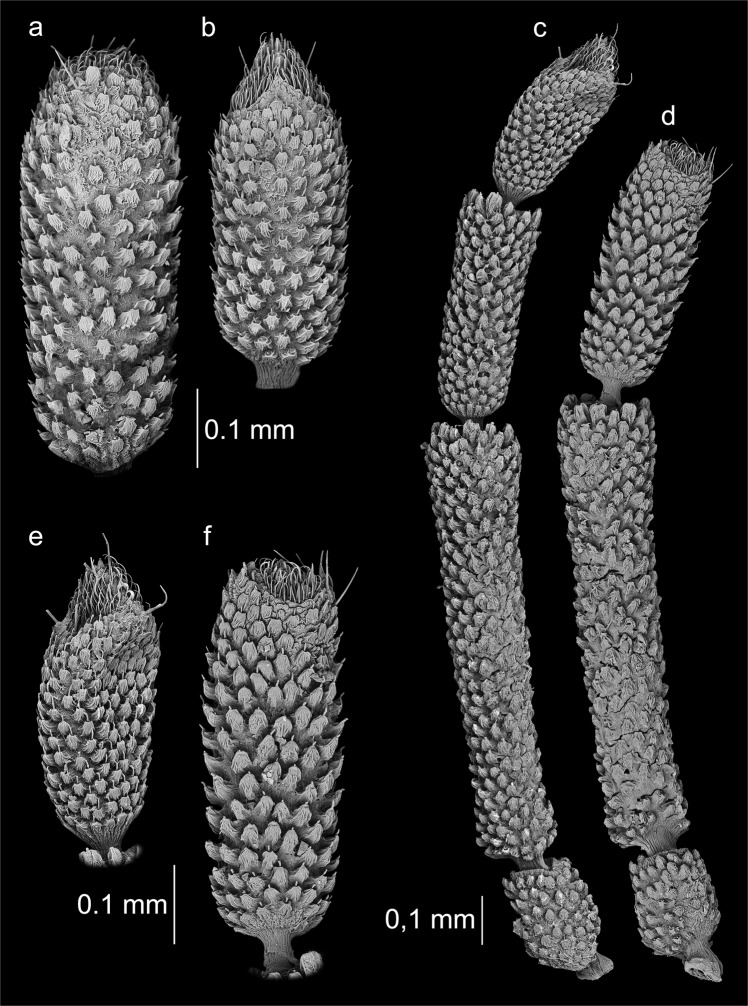
Antennae of the studied specimens. *Aradus betulinus*: (**a**) third (last) segment of an oligomeric antenna; (**b**) fourth (last) segment of a properly developed antenna; *Aradus corticalis*: (**c**) a properly developed antenna; (**d**) an oligomeric antenna; (**e**, **f**) the last segments at a higher magnification.

**Figure 4:**
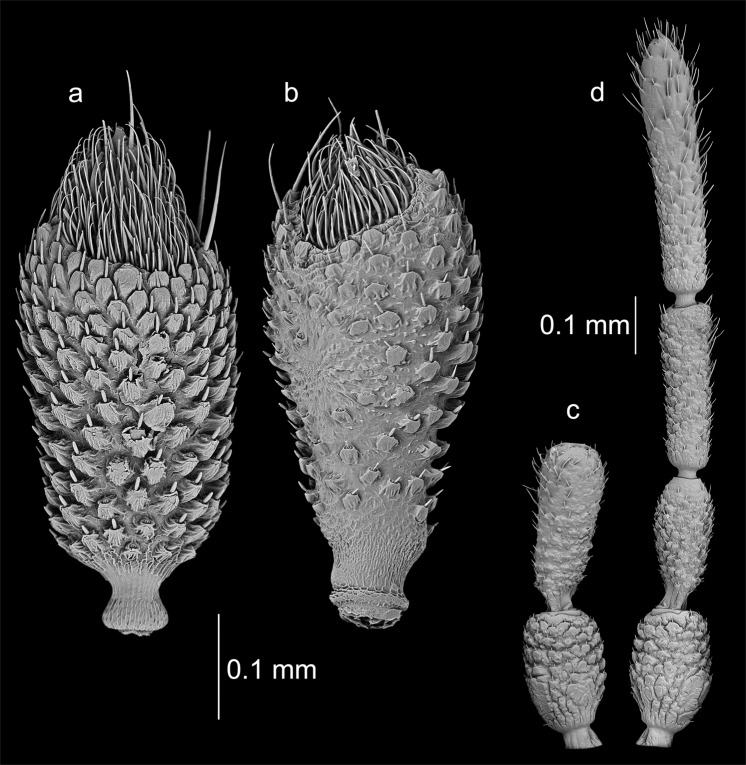
Antennae of the studied specimens. *Aradus depressus*: (**a**) fourth (last) segment of a properly developed antenna; (**b**) third (last) segment of an oligomeric antenna; *Aneurus laevis*: (**c**) an oligomeric antenna; (**d**) a properly developed antenna.

**Figure 5:**
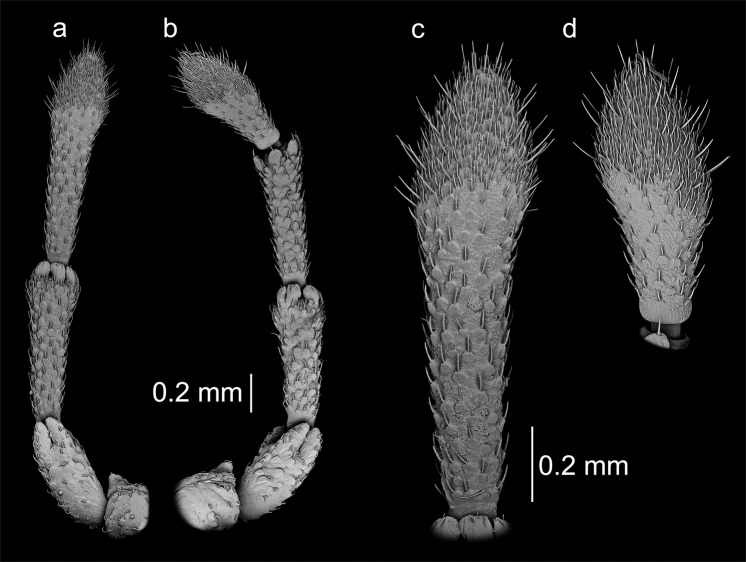
Antennae of *Brachyrhynchus membranaceus*: (**a**) an oligomeric antenna; (**b**) a properly developed antenna; (**c**,**d**) the last segments at a higher magnification.

Teratology was observed in 14 specimens belonging to eight species. In all of the cases, the antennal malformation could be classified as unilateral oligomery. A detailed list of the morphological malformations (observed using stereoscopic microscopy) of all of the species is presented below (Figs 1 and 2). The antennae of five specimens were additionally analyzed in SEM. Only specimens with undamaged antennae, which permitted the regeneration of sensory organs to be determined, were studied (Figs 3–5).

*Aradus betulae*, ♀

- left antenna oligomeric, three-segmented – 1^st^ segment normal, 2^nd^ only slightly shortened, 3^rd^ morphologically similar to the 4^th^ segment, but slightly shorter (Fig. 1a).
- right antenna normal.

*Aradus betulae*, ♂

- left antenna normal;
- right antenna oligomeric, three-segmented – 1^st^ segment normal, 2^nd^ only slightly thickened, 3^rd^ morphologically similar to the 4^th^ segment and close to it in size (Fig. 1b).

*Aradus betulinus*, ♂

- left antenna normal.
- right antenna oligomeric, three-segmented – 1^st^ segment normal, 2^nd^ normal, 3^rd^ morphologically similar to the 4^th^ segment, but it was much larger (Fig. 1c).

*Aradus betulinus*, ♂

- left antenna oligomeric, three-segmented – 1^st^ segment normal, 2^nd^ normal, 3^rd^ to some extent morphologically similar to 4^th^ segment, but it is elongate (Figs 1d and 3a,b). The sensory cone was regenerated to some extent – it was more flattened at the top; however, all of the types of sensilla were present (Fig. 3a,b).
- right antenna normal.

*Aradus betulinus*, ♀

- left antenna normal.
- right antenna oligomeric, three-segmented – 1^st^ segment normal, 2^nd^ only slightly shortened, 3^rd^ morphologically similar to the 4^th^ segment, but it was wider in the apical part (Fig. 1e).

*Aradus conspicuus*, ♀

- left antenna oligomeric, three-segmented – 1^st^ segment normal, 2^nd^ normal, 3^rd^ to some extent morphologically similar to the 4^th^ segment, but it was more elongated (Fig. 1f).
- right antenna normal.

*Aradus conspicuus*, ♂

- left antenna normal.
- right antenna oligomeric, three-segmented – 1^st^ segment normal, 2^nd^ only very slightly shortened, 3^rd^ morphologically similar to the 4^th^ segment, but it was elongate (Fig. 1g).

*Aradus corticalis*, ♀

- left antenna normal.
- right antenna oligomeric, three-segmented – 1^st^ segment normal, 2^nd^ normal, 3^rd^ to some extent morphologically similar to the 4^th^ segment, but it was elongate and a bit different in shape, more massive (Figs 1h and 3c–f). The sensory cone was regenerated to some extent – it was more flattened at the top; however, all of the types of sensilla were present (Fig. 3c–f).

*Aradus corticalis*, ♂

- left antenna oligomeric, three-segmented – 1^st^ segment normal, 2^nd^ normal, 3^rd^ morphologically similar to the 4^th^ segment, but it was larger and wider in the apical part (Fig. 1i).
- right antenna normal.

*Aradus depressus*, ♀

- left antenna normal.
- right antenna oligomeric, three-segmented – 1^st^ segment normal, 2^nd^ very slightly elongate, 3^rd^ morphologically similar to the 4^th^ segment (Figs 2a and 4a,b). The sensory cone was regenerated to some extent – it was smaller; however, all of the types of sensilla were present (Fig. 4a–b).

*Aradus depressus*, ♂

- left antenna oligomeric, two-segmented – 1^st^ segment normal, 2^nd^ morphologically similar to the 4^th^ segment, but it was larger and a bit different in shape, clavate (Fig. 2b).
- right antenna normal.

*Aradus erosus*, ♂

- left antenna normal.
- right antenna oligomeric, three-segmented – 1^st^ segment normal, 2^nd^ only very slightly shortened and thickened, 3^rd^ to some extent morphologically similar to the 4^th^ segment, extended in the middle part (Fig. 2c).

*Aneurus laevis*, ♀

- left antenna oligomeric, two-segmented – 1^st^ segment normal, 2^nd^ enlarged, elongate and cylindrical in shape (Figs 2d and 4c,d). Regeneration of the sensilla occurred to a small extent. On the second segment, sensilla chaetica occurred on the third and especially on the fourth segment of the properly formed antenna; however, they were much less abundant and were smaller (Fig. 4c,d).
- right antenna normal.

*Brachyrhynchus membranaceus*, ♂

- left antenna oligomeric, three-segmented – 1^st^ segment normal, 2^nd^ slightly elongate, 3^rd^ morphologically similar to the 4^th^ segment, but it was significantly elongate (Figs 2e and 5a–d). The regeneration of the distal part of the 4^th^ segment, which was characterized by numerous sensilla, occurred completely in terms of the number, distribution and types of sensilla (5a–d).
- right antenna normal.

**Figure 6:**
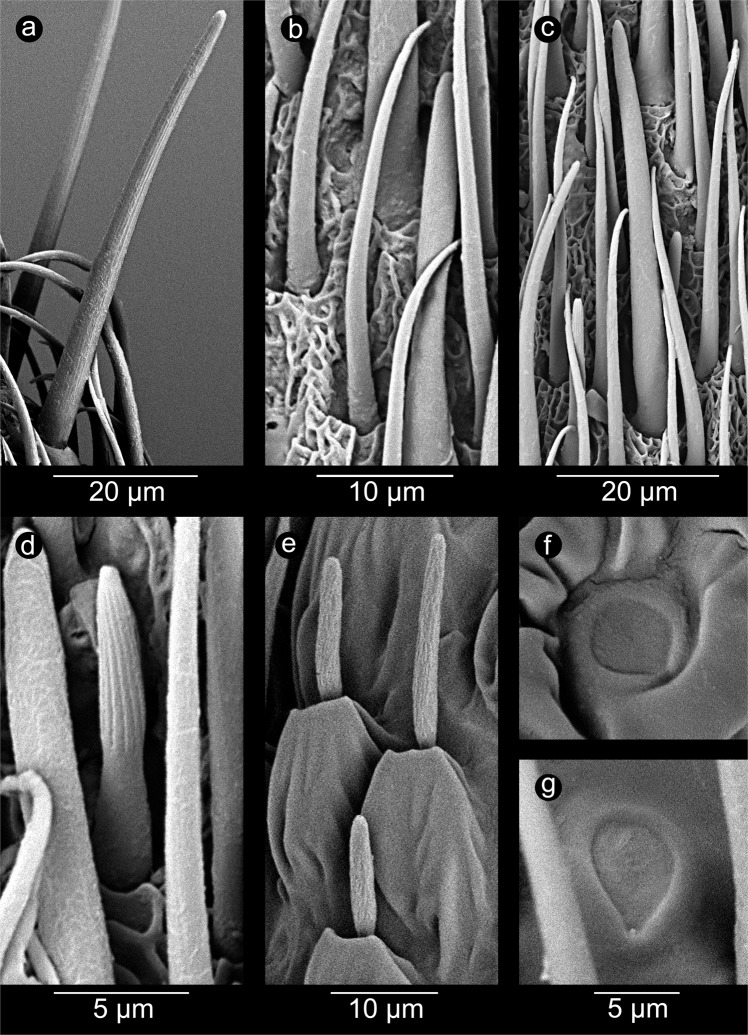
Particular types of sensilla (in the center of each figure). *Aradus betulae*: (**a**) chaetic sensillum Ch1; (**b**) trichoid sensillum T1; (**c**) trichoid sensillum T3; (**d**) basiconic sensillum B2; *Aradus depressus*: (**e**) styloconic sensilla S; (**f**) campaniform sensillum C; *Brachyrhynchus membranaceus*: (**g**) campaniform sensillum C.

**Figure 7:**
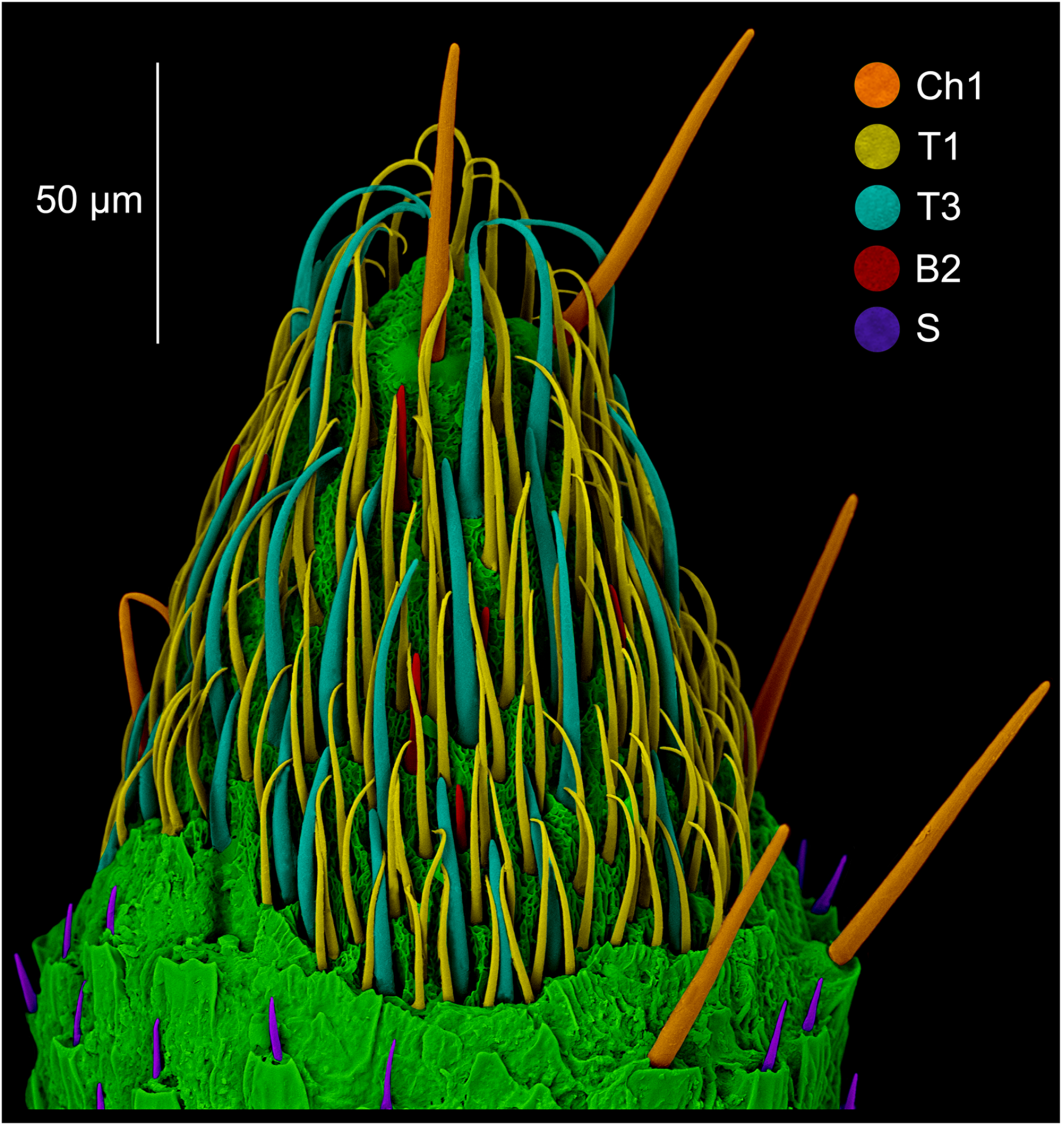
Distribution of sensilla on the properly developed sensory cone of *Aradus betulae*: Ch1 – sensilla chaetica; T1, T3 – trichoid sensilla; B2 – basiconic sensilla, and below its: S – styloconic sensilla.

The detailed morphology of particular observed types of sensilla was presented in the Fig. 6. The distribution and diversity of sensilla on sensory cone on the example of *Aradus* *betulae* was shown on Fig. 7. A total of six types of sensilla were found in examined specimens: sensilla chaetica Ch1, sensilla trichoidea T1 (Fig. 6b) and T3 (Fig. 6c), basiconic sensilla B2 (Fig. 6d), styloconic sensilla S (Fig. 6e) and campaniform sensilla (Fig. 6f,g) which had not been previously noted in Aradidae. This type of sensillum has been observed in two species: *Aradus depressus* (both properly formed and teratological antennae) as well as *Brachyrhynchus membranaceus*, only on normal antennae. In case of *A*. *depressus*, single campaniform sensillum just below the sensillar cone has been observed. It had the form of a circle with a diameter of 4.8 μm (Fig. 6f). A single sensillum was also found in *B*. *membranaceus*. It had a teardrop shape 6.8 μm long and 5 μm wide (Fig. 6g).

## Discussion

Our research confirmed the presented hypothesis – antennal teratology is not a rare phenomenon in the family Aradidae. Teratological (unilateral oligomeric) antennae were observed in 14 of the 476 studied specimens (2.94%). Oligomery was unilateral in all of the specimens. In studies within Tingidae, one teratological case was found per 40 collected specimens (2.5%)^36^. In extensive studies on Lygaeidae *s*.*l*. (Lygaeidae, Heterogastridae, Oxycarenidae, Rhyparochromidae), there were 60 specimens with an antennal malformation per 4,000 examined insects, which was a frequency of teratology of 1.5%^14^. Štusák and Stehlik reported that a variation in the incidence of teratology may also depend on systematic and morphological dispositions, and also may depend on the possibility of suffering from an injury, which is connected with the ecological and biological properties of a species^15^. In the groups of Heteroptera that are mobile and prefer less secure habitats and are thus exposed to more frequent injuries during the nymphal instars, anomalies occur more frequently (e.g. in representatives of the family Rhyparochromidae, which run on the soil surface) than in groups that are less mobile and that live in a more sheltered way, such as some species of the family Tingidae. However, the high incidence of antennal malformation in Aradidae that was found in this study contradicts this statement. Flat bugs live under the bark of decaying trunks^29^, which are places where they are relatively poorly exposed to injuries, and yet, as many as 2.94% of the examined specimens were characterized by antennal malformations. It may be, however, that this results from some unknown morphological properties or other features that affect the regeneration process. It is also possible that it is their cryptic lifestyle that allows survival of individuals with certain aberrations. If such aberrations do not significantly impede the sensory functions of the antennae, then living in a confined and secure environment with high food supply lower the selective pressure and aberrant individuals may have high chances of survival and reproduction. Similar life mode observed in other hemipterans, such as aphids of the genus *Stomaphis* lead to increased number of aberrations of antennae in dwarfish males^20^. The same process may be involved here – higher number of individuals of both sexes in a small space increases possibility of effective mating and reproduction, despite malformed antennae.

The regeneration of damaged antennae in the examined specimens occurred to various degrees. In eleven cases, the regenerated antennae were three-segmented, while two were two-segmented. In the first case, the second segment of the regenerated antenna in nine individuals was of a normal length and the third resembled a properly formed segment to some degree (Figs 1b,e and 2a,c). In some cases, compensatory regeneration occurred – the regenerated segments were larger than normal (Figs 1c,d,f–i, 3a–f and 5a,d). In the above-mentioned cases, individuals lost only one segment of an antenna. In a study of the regeneration of antennae after the removal (in first instar) of the distal segment in *Riptortus clavatus* (Alydidae), Ikeda-Kikue and Numata found that the gradual regeneration of both the length and sensory organs of the shortened antenna occurred; however, it still remained three-segmented^27^. The important thing is that after the amputation of the distal (fourth) segment, not only the third (new distal) segment but also the second segment grew excessively. However, a situation in which these individuals could lose two or three segments is also possible. Wolsky indicated that after removal (in first instar) of two or three segments in the antennae of *Oncopeltus fasciatus*, the number of segments was always remained one less than in properly developed antennae, but that the segments grew longer and more massive than typical^23,27^. Another experiment, the object of which was *O*. *fasciatus*^26^ also showed that removing of the three segments of an antenna usually resulted in the regeneration of only single segment (despite a small percentage of the regenerates consisting of two segments and several consisting of a segment with only a partial intersegmental membrane), which was incorrectly long but presented a set of sensory hairs (sensilla) that is typical of the two distal segments of normal antennae.

In two specimens (Fig. 1a,e), the second segment of the regenerated antenna was smaller than normal, and therefore it had to be lost. In the next two specimens (Figs 2b,d and 4c,d), there was a loss of three segments and only one was regenerated. The first was normally shaped in all of the antennae.

Aradidae representatives have the majority of their sensilla types on the last segment of the antennae. This is particularly strong in representatives of the genus *Aradus* (Aradinae), in which the sensilla (except styloconic) at the distal part of the fourth segment of the antennae (Fig. 7) – the “sensillary cone” are arranged^30^, which is not much weaker in the case of *Brachyrhynchus membranaceus* (Mezirinae), but is poorly developed in *Aneurus laevis* (Aneurinae) in which chaetica and trichoid sensilla are found on the whole third and fourth segments. During the study, five out of six types of sensilla recorded so far in flat bugs (in fact only one species has been studied in this respect – *Aradus corticalis*) were found^30^. In addition, campaniform sensilla was first discovered in this group of Heteroptera (Fig. 6). The presence of basiconic sensilla (B1) has not been confirmed.

Observations using scanning electron microscope showed that functional regeneration (sensory organs) occurred to varying degrees. In *Brachyrhynchus membranaceus*, the sensilla regenerated to a large extent – in terms of type, size and distribution (Fig. 5a–d). In other cases, although all of the types of sensilla regenerated, their arrangement was different than the normal one (Figs 3a–d and 4a,b). In the case of *Aneurus laevis*, regeneration was very poor (Fig. 4c,d). There can be various reasons for the different levels of regeneration – the number of instars, the time before the ecdysis, the degree and type of injury, ecological factors and the condition of the individual^15^. When specimens that are caught in the field are examined, their determination is difficult.

In order to better understand the phenomenon of antennae teratology in Heteroptera, further studies are necessary. Because antennal malformation have not been found or have been observed very rarely in many groups – research on individuals that are collected in the environment are important. For example, it cannot be excluded that the regeneration in Cimicomorpha could, to a certain degree, have escaped the attention of specialists, since in most families that belong to this group, the antennae are extremely slender, and therefore, antennal regenerates are less apparent. This hypothesis was confirmed by recent reports on the occurrence of teratological antennae in the family Miridae^25,37^. Within this infraorder, stronger antennae primarily occur in some genera of the family Tingidae and in their case, teratology was frequently found^15^. On the other hand, studies of the regeneration possibilities that are dependent on the number of instars, the time before the ecdysis or the degree and type of injury (therefore research that can only be carried out under controlled laboratory conditions) are necessary. In addition, the problem of compensatory regeneration and specifically the maintenance of sensory functions of the antennae are interesting. Unfortunately, knowledge about the sensillar structures in most Heteroptera groups is very poor, which makes analyzing them quite difficult. Another problem is the various nomenclature used to describe each type of sensillum [e. g.^30,35,38,39^]. Due to the limited research material as well as different research purpose, it was not decided to consider this issue in this paper. Undoubtedly, however, there is a need for critical revision and standardization of nomenclature regarding sensory organs of insects.

## Data Availability

All data generated or analyzed during this study are included in this published article.
